# Understanding Lamotrigine’s Role in the CNS and Possible Future Evolution

**DOI:** 10.3390/ijms24076050

**Published:** 2023-03-23

**Authors:** Bárbara Costa, Nuno Vale

**Affiliations:** 1 OncoPharma Research Group, Center for Health Technology and Services Research (CINTESIS), Rua Doutor Plácido da Costa, s/n, 4200-450 Porto, Portugal; 2 CINTESIS@RISE, Faculty of Medicine, University of Porto, Alameda Professor Hernâni Monteiro, 4200-319 Porto, Portugal; 3 Department of Community Medicine, Information and Health Decision Sciences (MEDCIDS), Faculty of Medicine, University of Porto, Rua Doutor Plácido da Costa, s/n, 4200-450 Porto, Portugal

**Keywords:** LTG, anti-epileptic drugs, central nervous system, cancer research, GABAergic neurotransmission

## Abstract

The anti-epileptic drug lamotrigine (LTG) has been widely used to treat various neurological disorders, including epilepsy and bipolar disorder. However, its precise mechanism of action in the central nervous system (CNS) still needs to be determined. Recent studies have highlighted the involvement of LTG in modulating the activity of voltage-gated ion channels, particularly those related to the inhibition of neuronal excitability. Additionally, LTG has been found to have neuroprotective effects, potentially through the inhibition of glutamate release and the enhancement of GABAergic neurotransmission. LTG’s unique mechanism of action compared to other anti-epileptic drugs has led to the investigation of its use in treating other CNS disorders, such as neuropathic pain, PTSD, and major depressive disorder. Furthermore, the drug has been combined with other anti-epileptic drugs and mood stabilizers, which may enhance its therapeutic effects. In conclusion, LTG’s potential to modulate multiple neurotransmitters and ion channels in the CNS makes it a promising drug for treating various neurological disorders. As our understanding of its mechanism of action in the CNS continues to evolve, the potential for the drug to be used in new indications will also be explored.

## 1. Overview of Antiepileptic Drugs

Anti-epileptic drugs (AEDs) or anticonvulsants are used to prevent or treat seizures; they work by changing the activity of certain neurotransmitters in the brain responsible for generating and spreading seizures. By changing the activity of these neurotransmitters, AEDs can prevent the abnormal electrical activity in the brain that causes seizures [1]. AEDs do not cure epilepsy but rather help manage the condition. Even when seizures are controlled with medication, it is essential to continue taking AEDs as prescribed since sudden withdrawal of AEDs can lead to seizures and other complications [2]. There are many different types of AEDs available, and the choice of medication depends on the type of seizure a person is experiencing, their age, and other factors such as other health conditions or medications they may be taking [3]. Some AEDs, such as phenytoin and carbamazepine, block the repetitive activation of sodium channels [4], while lacosamide enhances their slow inactivation [5]. Drugs like ethosuximide and valproic acid block T-type calcium channels, while zonisamide blocks both N- and L-type calcium channels [6]. Lamotrigine employs a more complex mechanism of action involving the blocking of both sodium and N- and L-type calcium channels as well as the modulation of H-current [7,8,9]. Similarly, topiramate blocks sodium channels and alpha-amino-3-hydroxy-5-methyl-4-isoxazole propionic acid (AMPA) receptors and inhibits carbonic anhydrase [10]. Other AEDs work by enhancing gamma-aminobutyric acid (GABA)-A receptors, such as phenobarbital and benzodiazepines blocking N-methyl-D-aspartic acid (NMDA) receptors, as felbamate does [11], or opening neuronal potassium channels, which is the mechanism of action of ezogabine [12].

Categorizing seizures as partial or generalized is utilized to decide on the appropriate AED for treatment. Simple partial seizures cause neurological findings in specific brain areas without affecting awareness. In contrast, complex partial seizures lead to confusion brain areas. Generalized seizures affect the entire body and manifest as tonic-clonic, absence, or atonic seizures [13]. Monotherapy is the preferred treatment for seizures, but obtaining FDA approval as a monotherapy agent is challenging for newer AEDs [14]. Second-generation AEDs have demonstrated similar efficacy to older AEDs and are potentially better tolerated [15]. AEDs can cause adverse effects, many of which are mild and reversible. However, severe side effects include osteoporosis, Stevens-Johnson syndrome, and an increased risk of suicidal behavior. Long-term AED use may cause osteoporosis, so patients should supplement their diet with calcium and vitamin D and establish regular exercise routines [16,17]. Drug interactions between AEDs and other medications usually arise with older AEDs, leading to either rapid metabolism of other drugs or toxic levels by affecting hepatic enzymes [18].

**Figure 1:**
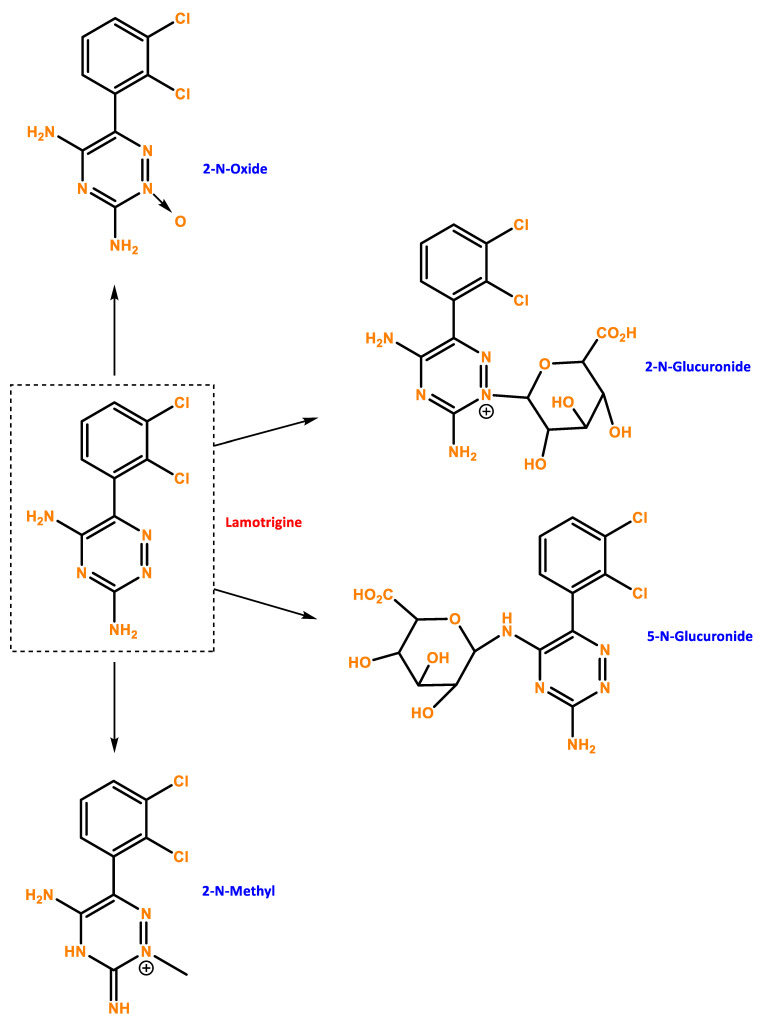
Chemical structure of LTG and possible metabolites. Developed with ChemBioDraw^®^ Ultra 13.0 ChemDraw^®^, a chemical drawing software.

Understanding the different mechanisms of action of AEDs is important because it allows healthcare professionals to choose the most appropriate treatment for each individual patient. It also helps in developing new drugs with more targeted mechanisms of action and fewer side effects [19]. In this review, we will be focusing on lamotrigine (LTG), a broad-spectrum AED that was initially found while researchers looked for phenytoin analogues with less potent anti-folate effects (Figure 1). Lamotrigine, which blocks sodium channels and inhibits the release of glutamate and aspartate, is effective in treating a range of seizures [2,20]. It shares structural similarities with medications that block dihydrofolate reductase (DHFR) [21]. LTG is similarly effective as carbamazepine (CBZ) or phenytoin (PHT), but it is better tolerated than valproic acid (VLP) and has a more favorable adverse drug reaction (ADR) profile [22].

### 1.1. Therapeutic Use and Drug Interaction

Lamotrigine is considered a first-line treatment for several types of epilepsy, including generalized and focal seizures, and it is often used in combination with other anti-epileptic medications [23]. Currently, LTG is FDA-approved for several indications in the central nervous system (CNS). The specific indications for using lamotrigine as adjunctive therapy in epilepsy include the treatment of partial seizures, primary generalized tonic-clonic seizures, and generalized seizures of Lennox-Gastaut syndrome. In patients who are 16 years of age or older, lamotrigine can also be used as a monotherapy for the treatment of partial seizures. This is typically done in patients who are already receiving treatment with carbamazepine, phenobarbital, phenytoin, primidone, or valproate as a single anti-epileptic drug. In addition to its use in treating epilepsy, LTG is also used to treat bipolar disorder in patients who are 18 years of age or older. Specifically, it is used as a maintenance treatment for bipolar I disorder to delay the occurrence of mood episodes in patients treated for acute mood episodes with standard therapy [24]. The prescription for taking LTG will vary depending on the treatment indication, but typically the starting dose is low and gradually increased to achieve the desired therapeutic effect, as needed.

The optimal dosage of LTG varies depending on the patient population. In adults with epilepsy, the recommended starting dosage is 25 mg once daily, with gradual increases of 25–50 mg every one to two weeks until the desired therapeutic effect is achieved. The maintenance dosage is typically 100–200 mg per day [25]. In pediatric patients, the recommended starting dosage is 0.6 mg/kg per day, with gradual increases of 0.2–0.3 mg/kg per day every one to two weeks until the desired therapeutic effect is achieved [26]. The maintenance dosage for pediatric patients is typically 5–15 mg/kg daily. In adults with bipolar disorder, the recommended starting dosage is 25–50 mg per day, gradually increasing 25–50 mg per week until the desired therapeutic effect is achieved. The maintenance dosage is typically 200–400 mg per day [27]. It is important to note that LTG should be taken with caution in patients with liver or kidney dysfunction and in those who are pregnant or breastfeeding [21,28].

Although lamotrigine (LTG) is widely used, it can lead to significant medication-drug interactions. For instance, carbamazepine (CBZ) and phenytoin accelerate LTG’s metabolism, decreasing its serum half-life from 15–35 h to 8–20 h [29]. One of the most commonly used combinations is LTG and valproate (VPA), which has a proven synergistic effect. However, VPA can decrease LTG clearance by 54% in combination therapy and by 21% in triple therapy with CBZ, leading to skin rash and even fatal Stevens-Johnson syndrome. Additionally, CBZ and LTG usage in combination therapy may be associated with a higher incidence of neurotoxic adverse effects [30,31].

On the other hand, combining LTG with other broad-spectrum AEDs, such as levetiracetam (LEV) or tocopheryl phosphate mixture (TPM) may be feasible because these drugs have no effect on LTG clearance [32]. Lastly, it is essential to note that rifampicin, a potent inducer of several drug-metabolizing enzymes, including CYPs and uridine-diphosphate glucuronosyltransferase (UGT), can decrease LTG concentration [33]. Oral contraceptives, including estradiol, also dramatically lower serum/plasma levels of LTG, which is metabolized by the liver’s UGT enzymes, which are affected by natural estrogens such as estrogen and ethinyl estradiol. Due to this action, LTG clearance can significantly rise throughout pregnancy, though substantial interindividual variability exists [34]. Hemodialysis efficiently removes LTG and its metabolites from the blood, making it a potential treatment for LTG overdose [35].

The mechanism of action of LTG Is complex, as the inactivation of sodium channels is a process that occurs over at least two kinetic time courses: fast and slow. This drug primarily binds to the fast-inactivated state of sodium channels, as opposed to the slow-inactivated state [36]. This means that, in therapeutic concentrations and at relatively depolarized membrane potentials, LTG can effectively inhibit sodium currents by slowly binding to the fast inactivated state of sodium channels. This mode of action is similar to phenytoin. The slow binding rates of both LTG and phenytoin may explain why these drugs can effectively inhibit seizure discharges while sparing most normal neuronal activities [37]. Therefore, LTG has been shown to be an effective treatment for epilepsy with a favorable safety profile due to its selective inhibition of fast inactivated state sodium channels [38]. LTG’s mechanism of action, compared to other AEDs, may provide advantages in treating certain types of seizures; see Table 1. LTG works by blocking voltage-dependent sodium channels [39], reducing calcium currents [40], and inhibiting the release of certain neurotransmitters in the brain, such as glutamate and aspartate [41], contributing to its broad efficacy. This blockage only happens during seizure activity, reducing side effects during non-seizure times; however, its mechanism is not fully understood [42]. Other neurotransmitters, including serotonin, norepinephrine, and dopamine, seem not to be significantly affected by LTG [43]. This helps stabilize the electrical activity in the brain and reduce seizures [16]. In summary, the mechanism of action may provide advantages in treating seizures associated with glutamate excitotoxicity. However, other AEDs with different mechanisms of action may be more effective in treating other types of seizures, such as those associated with GABA imbalances or calcium channel dysfunction. Additionally, lamotrigine is less likely to cause sedation or cognitive impairment compared to some other anti-epileptic drugs, which makes it effective for the treatment of certain mood disorders in addition to epilepsy [44].

Different reviews try to compare the efficacy of various anti-epileptic drugs. Campos MSA et al. compared the monotherapy treatment of generalized epileptic seizures, including valproate, lamotrigine, phenytoin, carbamazepine, topiramate, levetiracetam, and phenobarbital. Lamotrigine demonstrated the highest probability of leaving patients seizure-free (61%) in the treatment of generalized tonic-clonic, tonic, and clonic seizures, followed by levetiracetam (47%) and topiramate (44%). However, valproate and ethosuximide were found to be the best options for the treatment of absence seizures, with both drugs more likely than lamotrigine to keep the patient seizure-free. Overall, lamotrigine was found to be as effective as valproate for treating generalized seizures [45]. A different study aimed to compare the effectiveness and tolerability of oxcarbazepine and lamotrigine monotherapies in pediatric patients with focal epilepsy. The study found that lamotrigine was more effective than oxcarbazepine in terms of seizure outcome after 12 months. It can be selected as a first-line monotherapy for treating pediatric focal epilepsy, regardless of MRI findings or development. The study also found that both drugs showed similar tolerability in terms of retention rate, drug discontinuation, and adverse effects. However, the retention rate was higher in patients with MRI abnormalities or developmental delays in the lamotrigine group. The study suggests that the choice of lamotrigine as a first-line monotherapy for treating pediatric focal epilepsy can lead to a seizure-free state [46]. Finally, according to a recent study from the Cochrane review, the effectiveness of 12 anti-epileptic drugs (AEDs) was compared for the treatment of focal onset seizures or generalized tonic-clonic seizures in children and adults. The study found that LTG was the most effective drug for individuals with focal onset seizures, performing better than most other treatments in terms of treatment failure for any reason and due to adverse events, including the other first-line treatment, CBZ. Lamotrigine was found to be as effective as levetiracetam for any treatment failure outcome. For people with generalized-onset seizures, the evidence was more limited, and sodium valproate was found to be the most effective first-line treatment. Still, there were no differences between sodium valproate, lamotrigine, or levetiracetam in terms of treatment failure. The study utilized an individual participant data (IPD) and network meta-analysis (NMA) review approach. It investigated the time to treatment failure, the time to achieve a twelve month remission, the time to achieve a six month remission, and the time to the first seizure post-randomization as its primary and secondary outcomes [47].

In summary, these studies have shown that lamotrigine is an effective anti-epileptic drug for treating generalized tonic-clonic, tonic, and clonic seizures, performing as well as valproate in this regard. However, for treating absence seizures, valproate and ethosuximide are better options. Lamotrigine is also an effective first-line monotherapy for treating pediatric focal epilepsy, regardless of MRI findings or development, and has similar tolerability to oxcarbazepine. Finally, the Cochrane review found that lamotrigine was the most effective drug for treating focal-onset seizures, performing better than most other treatments.

When it comes to comparing different anti-epileptic drugs, it is important to do so carefully. This is because these drugs have different mechanisms of action and clinical experiences. Evidence-based medicine is crucial in characterizing anti-epileptic drugs for optimal decision-making, especially with the introduction of both traditional and new drugs in the field of focal epilepsy. However, clinicians should not overlook their personal experience and patient-related factors such as age, comorbidity, adverse effects, and economic state when deciding on the best treatment for their patients. It is, therefore, essential to conduct more meta-analyses to study the differences and successfully compare drugs such as lamotrigine to others in their class. This will help guide treatment decisions in specific populations and ensure the best possible outcomes for patients.

**Table 1:** Comparison of LTG with other anti-epileptic drugs.

Feature	Lamotrigine	Other Anti-Epileptic Drugs
Mechanism of action	Preferentially inhibits sodium channels by binding to the fast inactivated state, which slows the recovery of the channel to its active state. This results in the reduction of neuronal excitability and decreases the likelihood of seizures.	Other AEDs, like valproic acid, increase the inhibitory neurotransmitter GABA in the brain, while others, such as topiramate, modulate glutamate receptors. The differences in the mechanisms of action among AEDs suggest that the optimal treatment for each person with epilepsy may differ depending on the type of seizures they experience and their individual response to the medication [1,48].
Side effects	Rashness, nausea, dizziness, and headache	Phenytoin, phenobarbital, carbamazepine, and oxcarbazepine also have the potential for serious allergic reactions and should be avoided in patients who have had previous serious or multiple allergic drug reactions. Valproate and lamotrigine can cause or exacerbate tremor and are not the drugs of choice for patients with essential tremor [49]. Carbamazepine and its derivative, oxcarbazepine, can cause hyponatremia, which is most common in the elderly treated with antihypertensives such as diuretics or angiotensin-converting enzyme inhibitors. Additionally, old hepatic enzyme-inducing medications like phenytoin, phenobarbital, and carbamazepine, and long-term treatment with valproate, can contribute to osteoporosis, particularly in postmenopausal women or immobilized patients with epilepsy and severe encephalopathy, and should be avoided in these patients [50]. When started gradually, lamotrigine is well tolerated, but when given in greater dosages or otherwise, it causes drowsiness and nausea. Up to 5% of patients can experience a rash, which is frequently brought on by a quick titration. In 1% of cases, a severe rash, which is more frequent in children using VPA, can progress into the uncommon and potentially lethal Steven-Johnson syndrome. Ataxia, diplopia, headache, tremor, blood dyscrasia, gastrointestinal discomfort, psychosis, somnolence, insomnia, and lesser hypersensitivity reactions are some of the more adverse effects [21]
Efficacy	Effective for treating bipolar disorder and certain types of epilepsy	Sodium channel blockers like carbamazepine, oxcarbazepine, and phenytoin may be ineffective or even exacerbate generalized epilepsy (GE)-related seizures. The reason why one sodium channel blocker is effective against GE and others are not, remains unknown [1,32].

The reason lamotrigine has broader anti-seizure effects than other sodium channel blockers like phenytoin is unknown. However, LTG has a good safety profile. Skin rash is the most commonly reported side effect. To lower the chance of a skin rash, slow dose titration is recommended. Other side effects include headaches, nausea, dizziness, and ataxia. Although rare, serious idiosyncratic liver injuries may occur. When used as prescribed, LTG is generally safe and effective for treating CNS disorders. Nevertheless, further research is needed to understand its mode of action and possibly develop new indications for use.

### 1.2. Additional Mechanism of Action of Lamotrigine

In addition to its established mechanisms of action, LTG has been shown to modulate HCN channels and inhibit the release of glutamate, as well as possess antioxidant and neuroprotective properties, all of which contribute to its efficacy in treating these disorders and its potential for use in other conditions (Table 2). HCN channels, also known as hyperpolarization-activated cyclic nucleotide-gated channels, are ion channels that are responsible for generating the pacemaker currents in neurons and cardiac cells. HCN channels are activated by hyperpolarization and cyclic nucleotides, and they play a critical role in regulating the excitability of neurons and the heart [51]. LTG has been shown to modulate HCN channels in a number of ways [52]. First, it can directly block the HCN channels, thereby reducing the pacemaker current and slowing down the firing rate of neurons [53]. Second, it can increase the sensitivity of HCN channels to cyclic nucleotides, which can also reduce neuronal excitability [51,53]. The modulation of HCN channels by lamotrigine may impact disease outcomes and potential side effects. For example, the reduction of neuronal excitability by lamotrigine may be beneficial in the treatment of epilepsy and bipolar disorder, where excessive neuronal activity can lead to seizures and mood instability [53]. Still, on the other hand, it can inhibit pacemaker currents in the heart.

Furthermore, the modulation of HCN channels by lamotrigine may impact other brain functions beyond epilepsy and bipolar disorder, such as learning and memory, sleep, and pain. The HCN channels are widely expressed in the brain and are involved in a range of physiological processes. Therefore, the effects of lamotrigine on HCN channels may contribute to the cognitive and behavioral side effects that are commonly observed with this drug. Interestingly, HCN channels are regulated by calcium ions, which can activate intracellular signaling pathways that are important for synaptic plasticity, learning and memory, and other brain functions. HCN channels can also allow calcium ions to enter the cell, which can have a potent effect on intracellular signaling and gene expression [54]. Lamotrigine modulates HCN channels and has been shown to affect intracellular calcium signaling by inhibiting the release of calcium ions from intracellular stores and reducing the influx of calcium ions through voltage-gated calcium channels (VGCCs). The reduction of calcium influx can influence the calcium-dependent signaling pathways that are activated by HCN channels [53,55]. Further research is needed to fully understand the mechanisms underlying the interaction between HCN channels, calcium ions, and lamotrigine, and to develop new therapies for neurological and psychiatric disorders that target these pathways.

Another of its mechanisms involves inhibiting the release of glutamate, a neurotransmitter that contributes to neuronal excitability (Table 2). Multiple studies have demonstrated a decrease in glutamate levels in brain tissue and synaptic fluid following LTG treatment [56]. LTG has been found to modulate the activity of the inhibitory neurotransmitter GABA, leading to a decrease in neuronal excitability [7]. Several studies have reported an increase in GABA levels in brain tissue and synaptic fluid following LTG treatment, as well as a decrease in seizure activity. In addition, LTG has been shown to increase the release of the inhibitory neurotransmitter GABA, resulting in further reduction of excitability that leads to seizures. Although the effects of GABA modulation by LTG are not particularly strong, they occur consistently even outside of seizure activity. They may contribute to some of the psychotropic effects of LTG [57]. While lamotrigine can produce neurobehavioral toxicity in some individuals, it is generally well tolerated [58]. Several studies have also shown that lamotrigine has positive effects on psychological health that are unrelated to its effects on seizure frequency and intensity. Lamotrigine has been shown to have favorable impacts on patient perceptions of quality of life when compared directly to other drugs frequently used to treat epilepsy, such as carbamazepine and phenytoin. Lamotrigine has advantages beyond its usage in treating epilepsy and bipolar disorder, according to findings from two blinded studies and many open trials for individuals with severe mental disability and refractory epilepsy [59]. Overall, while lamotrigine was originally developed for the treatment of epilepsy, its unique mechanism of action and favorable effects on psychological well-being have made it a promising option for the management of bipolar disorder and other conditions [60].

Moreover, LTG has also been shown to have antioxidant properties (Table 2), which contribute to its neuroprotective effects by reducing oxidative stress and increasing cell viability [61]. Several studies have investigated the impact of LTG on oxidative stress, with malondialdehyde (MDA) levels and concentrations of glutathione (GSH), catalase (CAT), and superoxide dismutase (SOD) used as measures of stress intensity. One study evaluated the cognitive functions and oxidative stress in rat brains during chemically induced seizures using pentylenetetrazol (PTZ). Results showed that LTG administration in the PTZ-kindled epileptogenesis group significantly reduced MDA levels and increased GSH, SOD, and CAT activities in homogenized whole brain samples as compared to the carbamazepine-treated group. This demonstrates the potential of LTG to counteract oxidative stress in the brain and promote better cognitive outcomes [62]. Lastly, LTG has been demonstrated to reduce the activation of microglia, the resident immune cells of the central nervous system, which play a key role in the initiation and maintenance of neuroinflammation [63]. Animal models of neuroinflammation have shown that LTG can help reduce the severity of the inflammatory response and improve the outcome of the disease.

LTG’s ion channel effects are more crucial for anti-seizure effects and less so for mood-stabilizing effects. The exact mechanism by which LTG acts as a mood stabilizer remains a mystery, but its neuroprotective and anti-glutaminergic effects are potential explanations [64]. Although LTG’s effects overlap with those of other anti-epileptic and mood-stabilizing drugs, its unique clinical profile sets it apart.

**Table 2:** Known mechanisms of LTG action.

Mechanism of Action	Explanation	Evidence	References
Inhibition of glutamate release	LTG is believed to reduce the release of the neurotransmitter glutamate in the brain, leading to decreased excitability of neurons.	Multiple studies have demonstrated a decrease in glutamate levels in brain tissue and synaptic fluid following LTG treatment.	[65,66]
Voltage-dependent block of sodium channels	LTG has been shown to inhibit the opening of voltage-gated sodium channels, reducing the spread of electrical activity in neurons.	In vitro and in vivo studies have demonstrated a reduction in sodium channel activity following LTG treatment.	[8,67]
Modulation of GABA transmission	LTG has been suggested to increase the activity of the inhibitory neurotransmitter GABA, leading to decreased excitability of neurons.	Studies have shown an increase in GABA levels in brain tissue and synaptic fluid following LTG treatment and reduced seizure activity.	[68]
Antioxidant properties	LTG has been shown to have antioxidant properties, which may contribute to its neuroprotective effects.	In vitro and in vivo studies have demonstrated a reduction in oxidative stress and increased cell viability following LTG treatment.	[69,70]
Inflammation	Microglia, the resident immune cells of the central nervous system, play a key role in the initiation and maintenance of neuroinflammation. LTG has been demonstrated to reduce the activation of microglia, which may help prevent or reduce neuroinflammation.	Animal models of neuroinflammation that LTG can help reduce the severity of the inflammatory response and improve the outcome of the disease.	[63]

Lamotrigine is a medication that has demonstrated its effectiveness both for treating epilepsy and as a mood stabilizer, but its mechanism of action remains partially understood. Unlike other medications such as lithium or valproic acid, lamotrigine does not reduce the expression of protein kinase C or MARCKS, suggesting that it utilizes different intracellular mechanisms for long-term changes in neurobiology [71]. Moreover, lamotrigine has been found to inhibit the release of glutamate, similar to lithium, which may be linked to mood stabilizing or antidepressant effects. In addition to its conventional uses, lamotrigine has shown promise in treating several other CNS disorders. Studies have suggested that it could be a potential treatment for depression, anxiety, and other mood disorders. Furthermore, it has been investigated as a potential treatment for conditions such as Alzheimer’s disease and schizophrenia, but further research is necessary to confirm its efficacy in these areas [72]. LTG is a medication with a versatile range of therapeutic applications in the CNS. While it has already demonstrated efficacy in managing seizures and bipolar disorder, research suggests that it may also hold promise for the treatment of several other CNS disorders, including depression, anxiety, Alzheimer’s disease, and schizophrenia. However, to fully realize the potential of this medication, we need a more thorough understanding of its molecular mechanism of action and its associations with potential indications. In the meantime, LTG remains a valuable tool in managing seizures and bipolar disorder and a promising candidate for new indications. By continuing to investigate its mechanisms of action and uses, we can unlock new possibilities, not only for the treatment of CNS disorders but also for other diseases of interest [71,72].

### 1.3. Pharmacokinetics and Pharmacodynamics

Lamotrigine’s pharmacokinetics have been investigated in epileptic patients, healthy young and old volunteers, and volunteers with chronic renal failure. Lamotrigine is rapidly and completely absorbed after oral administration with negligible first-pass metabolism, and the absolute bioavailability is 98% [73]. Peak plasma concentrations occur anywhere from 1.4 to 4.8 h following drug administration. The plasma concentrations of lamotrigine increase in direct proportion to the dose administered over the range of 50–400 mg. The estimates of the mean apparent volume of distribution (Vd/F) of lamotrigine following oral administration ranged from 0.9 to 1.3 L/kg [74]. Lamotrigine is approximately 55% bound to human plasma proteins, and it is metabolized predominantly by glucuronic acid conjugation, with the major metabolite being an inactive 2-N-glucuronide conjugate. The elimination half-life and apparent clearance of lamotrigine depend on whether the patient is receiving enzyme-inducing drugs or not [75]. If stopping LTG is necessary, it should ideally be done gradually over two weeks. LTG withdrawal seizures are possible but are less likely if the medication is weaned off gradually rather than abruptly.

LTG is metabolized in the liver by glucuronidation and oxidation, being mainly metabolized by UGT enzymes in the body and excreted by the kidneys, with CYP enzymes not participating in its metabolism [76]. It has a half-life of 15–30 h, and it is often the first choice of anti-epileptic drug for women of childbearing age due to its safety. LTG passes through the blood-brain barrier to reach the epileptic center, and its efficacy is indicated by the concentration of LTG in the plasma [77]. Monitoring the plasma concentration of LTG is important for achieving satisfactory therapeutic effects and avoiding adverse reactions in patients with epilepsy. LTG has a dose-dependent effect and a slow onset of action. Its full therapeutic effect may only be seen in a few weeks or months [78,79]. Overall, the pharmacodynamics of lamotrigine are complex and varied, with potential effects on folate metabolism, kidney function, melanin-containing tissues, and the cardiovascular system, according to the FDA label [24].

The mechanism by which it crosses the blood-brain barrier is not completely understood, but studies have shown that organic cation transporters may play a role. There is conflicting evidence for the role of ABC transporters, such as P-glycoprotein and ABCG2, in LTG efflux across the blood-brain barrier. Polymorphisms in these genes have been associated with differences in LTG concentrations in plasma, which may explain differential drug response. LTG is extensively metabolized, with over 80% of the total dose recovered in the urine as glucuronides and primarily eliminated by ABCC transporters [80]. The relationship between gene polymorphisms and plasma LTG concentrations plays a crucial role in differential drug response. ABCC2 (MRP2) polymorphisms have been linked to drug resistance in various populations, suggesting that this transporter may be involved in drug efflux [81]. Despite inconsistent results from model systems, pharmacogenomic studies have provided important insights into the pharmacokinetics, pharmacodynamics, and mode of action of LTG. However, additional research is needed to gain a comprehensive understanding of LTG’s PK/PD and mechanism of action and enhance its therapeutic efficacy.

## 2. Lamotrigine Potential for Repurposing

### 2.1. Other CNS Disorders

LTG’s mechanisms of action and favorable safety profile have led to an investigation into its potential for repurposing in other central nervous system disorders (Table 3), and being chemically unrelated to other anti-epileptic drugs, it has been the subject of emerging research for its potential use in treating other CNS disorders. One area of interest is the management of neuropathic pain (NeP), a chronic pain caused by damage to the nervous system. Studies have suggested that LTG may effectively reduce pain intensity and improve quality of life in patients with neuropathic pain conditions such as diabetic neuropathy and post-herpetic neuralgia [82]. For specific cases, LTG is considered a second-line treatment. LTG (400 mg/day) has been shown to be effective in reducing pain in patients with trigeminal neuralgia who did not respond to carbamazepine in a double-blind placebo-controlled trial (14 patients) and an open-label study (11 of 15 patients). The recommended starting dose is 25 mg twice daily, increasing to 200–400 mg/day, but the required dose for pain relief varies widely [83]. LTG’s effectiveness has been investigated in several peripheral and central NeP diseases, but the findings have been conflicting. Negative findings have been found in research on severe diabetic neuropathy, studies on mixed NeP, and a single study on chemotherapy-induced NeP and spinal cord injury pain. On the other hand, research on HIV-related neuropathy, trigeminal neuralgia, and central post-stroke pain has found encouraging findings. However, these trials typically had small sample sizes and significant dropout rates [84]. Nowadays, LTG is occasionally used for painful HIV-related neuropathy; however, it is not frequently prescribed for neuropathic pain because it fits into the spectrum of medications used to treat different types of neuropathic pain [85]. Regarding fibromyalgia, a chronic pain disorder characterized by widespread musculoskeletal pain and tenderness, although lamotrigine has been suggested to improve pain and quality of life in individuals with fibromyalgia, there have been no studies specifically examining its efficacy in this condition. While fibromyalgia has a different cause than chronic neuropathic pain, it responds to similar treatments. Due to limitations in the number of available clinical trials, it is sometimes useful to consider fibromyalgia along with neuropathic pain. The most recent analysis we could find to elucidate on this matter was the 2013 Cochrane review on lamotrigine for chronic neuropathic pain and fibromyalgia in adults, which included twelve studies with 1511 participants, all with chronic neuropathic pain. The studies involved various conditions such as central post-stroke pain, chemotherapy-induced neuropathic pain, diabetic neuropathy, HIV-related neuropathy, mixed neuropathic pain, SCI-related pain, and trigeminal neuralgia. The review found that lamotrigine did not improve pain and was no different from a placebo, except that it caused more side effects. However, it is worth mentioning that only one study on SCI was included in the review, and in this trial, lamotrigine reduced neuropathic pain among incomplete SCI patients. Large, high-quality, long-duration studies reporting clinically useful levels of pain relief for individual participants provided no convincing evidence that lamotrigine is effective in treating neuropathic pain and fibromyalgia at doses of about 200–400 mg daily. Moreover, no studies were found testing lamotrigine in fibromyalgia [86].

Some studies have shown that LTG may reduce the severity of Post-Traumatic Stress Disorder (PTSD) such as re-experiencing and hyperarousal. Glutamate appears to be a key moderator in PTSD; therefore, anti-epileptic medications, such as LTG, which is a glutamate release inhibitor, may be useful in the management of PTSD symptoms. According to numerous research studies, the re-experiencing and avoidance/numbing symptoms of PTSD patients were improved by LTG [87,88]. In comparison to the placebo group, LTG treatment reduced PTSD symptoms in adults by about 50%, but obese individuals receiving LTG saw a significant weight loss [89]. Pham et al. demonstrated that using the LTG made it possible to observe a reduction in stress-related symptoms, including self-injurious behavior, as well as a reduction in body weight, effects anticipated and supported by our prior fundamental research findings in people and animals. Additionally, a recurrence of the localized symptoms occurred after decreasing the treatment dose of LTG, which is consistent with earlier findings [90,91].

The relationship between epilepsy and ADHD is unclear. ADHD symptoms, such as inattention, hyperactivity, and behavioral disturbances, can often be considered adverse effects of AEDs. A study of pediatric patients with a new diagnosis of epilepsy and ADHD found that LTG may help improve ADHD symptoms. However, the results of this study are limited due to the use of a Korean test used to measure ADHD symptoms. Carbamazepine and LTG may be more effective in treating both epilepsy and ADHD compared to other AEDs, as they can control seizures and enhance attention and behavior [92,93]. Moreover, adult ADHD patients with concurrent bipolar II disease have reported positive results on ADHD symptoms when using LTG [94]. Table 3 provides a summary of the potential uses of LTG for various CNS disorders and includes information on the clinical studies related to each of the potential uses (including the NCT identifier and references to relevant studies on the topic).

**Table 3:** Potential uses of LTG for other CNS diseases.

Condition	Potential Use of LTG	Clinical Studies (NCT Identifier)	References
Neuropathic pain	Used as a second- to fourth-line treatment. Occasionally used for painful HIV-related neuropathy.	NCT00913107 NCT00203229 NCT00295776 NCT00618241 NCT00243152 NCT04523935	[85]
PTSD	LTG may be useful as a primary psychopharmacological treatment for PTSD and may also be thought of as a supplement to antidepressant therapy in the treatment of PTSD.	NCT00571246	[89]
AHDH	Positive association with reducing ADHD probably due to achieving seizure freedom and normalizing electroencephalogram.	NCT01000402	[93]
Fibromyalgia	LTG can be effective in reducing pain and improving quality of life in individuals with fibromyalgia.	No studies found	[86]
Alcohol and ketamine addiction	LTG can be effective in reducing relapse to alcohol seeking in rats.	NCT01015586 NCT02556060	[95,96,97,98]
Migraine	Lamotrigine is a particular preventative therapy for migraine with aura.	Quality improvement and practice based research in neurology using the EMR-NCT02670161	[99]

Other conditions LTG is being studied for are alcohol or ketamine addiction and migraine. Preclinical studies have investigated the potential use of lamotrigine in the treatment of alcohol and ketamine addictions. Studies have suggested that lamotrigine also affects other neurotransmitter systems, such as serotonin and dopamine. As both glutamate and monoamines have been implicated in alcohol craving and relapse, researchers aimed to investigate the effects of LTG on alcohol seeking behavior and relapse-like drinking [95,100].

Research has demonstrated that LTG can decrease the rewarding effects of the drug ketamine and decrease the likelihood of relapse and craving for ketamine [101]. Some studies on healthy humans have also demonstrated a decrease in ketamine-induced dissociative symptoms with LTG pretreatment. However, a study on patients with depression did not find a stronger antidepressant effect with the combination of ketamine and LTG compared to ketamine alone, although only one ketamine infusion was administered in this study. In a case series, two bipolar patients experienced an antidepressant and anti-suicidal effect with the combination of ketamine and LTG. Available clinical studies on patients with mood disorders did not provide conclusive evidence that LTG reduces ketamine-induced dissociative symptoms. Overall, the results of the analyzed studies were not sufficient to answer the stated questions, but they provided directions for future research. Animal studies suggest a possible synergistic antidepressant effect of ketamine and LTG, and there is some evidence that LTG can reduce ketamine’s side effects and craving. However, more controlled studies on large groups of bipolar patients receiving multiple ketamine infusions combined with LTG treatment are needed, along with follow-up studies to investigate the effectiveness of the ketamine-LTG combination [95,96].

Migraine is another condition that has been studied in relation to lamotrigine [102]. While the exact mechanism of action is unclear, lamotrigine has been shown to be effective in reducing the frequency and severity of migraine attacks in some individuals. Many double-blind, randomized, placebo-controlled trials have demonstrated the effectiveness of AEDs like VPA and topiramate in preventing migraines [98]. However, some studies imply that lamotrigine is a particular preventative therapy for migraine with aura [97,99]. However, more research is needed to fully understand its effectiveness and how it works in this context.

While each of these conditions presents different symptoms, they share certain similarities, such as changes in the normal functioning of brain systems. By understanding the mechanism of action of LTG, we can gain insight into how this medication impacts these conditions and how it can be used to treat them. In epilepsy and bipolar disorder, LTG has been shown to have positive effects on reducing seizures and stabilizing mood swings, respectively. Similarly, in PTSD, LTG may help to alleviate symptoms by reducing hyperarousal and re-experiencing symptoms associated with the disorder. The mechanisms behind LTG’s efficacy in these CNS diseases are not fully understood, but research has suggested that its impact on the excitability of neurons may play a role. By continuing to study the mechanism of action of LTG, we can build a deeper understanding of the underlying causes of these conditions and develop more effective treatments for them. Overall, the potential for its use in these areas is promising, and further research is warranted to explore its potential benefits.

#### 2.1.1. The Relationship of LTG and the Blood-Brain Barrier (BBB)

LTG has been shown to have potential therapeutic uses in various CNS diseases, including bipolar disorder, epilepsy, neuropathic pain, and others. We know that the exact mechanism of action of LTG is not fully understood. One promising way to understanding the drug’s mechanism of action is by studying its relationship with the blood-brain barrier (BBB).

The BBB is a selectively permeable barrier that separates the circulating blood from the brain’s extracellular fluid. It is made up of tightly packed cells that restrict the passage of most substances, including ions, from the blood into the brain. This selective permeability ensures that the concentration of ions, such as sodium and potassium, is tightly regulated in the brain, which is essential for maintaining proper neuronal excitability. Any disruption in the BBB’s selective permeability can lead to an abnormal accumulation of ions in the brain and consequently affect neuronal excitability.

For example, in certain neurological disorders such as epilepsy, the BBB may become leaky, allowing excessive amounts of ions to accumulate in the brain, leading to abnormal neuronal activity and seizures. Additionally, certain medications, such as AEDs, can affect the BBB’s permeability [103], leading to changes in neuronal excitability and potentially influencing the effectiveness of the medication. Therefore, the permeability of the BBB is a critical factor in the efficacy of CNS medications, including LTG. For a medication to be effective, it must be able to penetrate the BBB and reach its target site in the brain. The permeability of the BBB is influenced by various factors, such as the size, charge, and lipophilicity of the molecule, which can affect its ability to cross the BBB and reach its target site in the CNS. LTG is known to have low permeability across the BBB, meaning that it can cross the BBB to some extent but not readily [104]. Despite this, LTG has been shown to be effective in treating various CNS conditions, indicating that it can reach its target site in the brain and exert its therapy [105].

Injuries to the CNS can disrupt the BBB, leading to inflammation and impaired brain function. Some evidence suggests that LTG may have a role in the repair of the BBB following CNS injury; however, its neuroprotective mechanism remains poorly studied [66]. Nevertheless, studies have shown that LTG can reduce inflammation, promote the repair of damaged blood vessels in the brain, and protect neurons while promoting their survival following CNS injury. However, further research is needed to understand these effects fully and determine their clinical implications. Recent studies have highlighted the BBB’s crucial role in the development and progression of various CNS diseases, including Alzheimer’s disease and certain types of cancer. By understanding the relationship between LTG and the BBB, researchers may explore the drug’s potential in treating these diseases as well. In summary, comprehending the relationship between LTG and the BBB is crucial for understanding the drug’s mechanism of action and exploring its potential therapeutic uses in other CNS diseases and cancer. Further research in this area may lead to the development of more effective treatments for these debilitating conditions.

#### 2.1.2. New Drug Delivery Systems for LTG

Despite its efficacy, using new drug delivery systems can improve LTG’s pharmacokinetic properties and brain targeting efficiency. The significant progress in LTG’s pharmacokinetic behavior and brain-targeting efficiency with new drug delivery systems can help improve its shortcomings. LTG has the major drawback of poor pharmacokinetics, causing low bioavailability and poor oral absorption, leading to reduced efficacy and a higher risk of side effects. However, advancements in drug delivery systems have demonstrated improvements in LTG’s pharmacokinetics. One example is using LTG-loaded PLGA nanoparticles for direct intranasal delivery, significantly improving pharmacokinetics and brain targeting effectiveness [106,107].

The use of liposomes also improved LTG pharmacokinetics. In a study, LTG-loaded liposomes were found to significantly improve LTG’s bioavailability and oral absorption compared to conventional oral administration [108]. This study aimed to increase LTG’s effectiveness against refractory epilepsy by creating LTG-loaded nanoliposomes (LTG-NL). Physico-chemical characterizations were performed on the prepared LTG-NL, and its distribution in mice was studied. The results showed that LTG-NL had the following characteristics: small, uniform, and spherical particles with high drug entrapment efficiency and controlled release. The distribution study indicated the brain selectivity of LTG-NL, and further evaluation confirmed its targeting capacity. The mechanism of entry into cells was determined to be through clathrin-mediated endocytosis and micropinocytosis, suggesting that LTG-NL could be an effective drug delivery system for insoluble drugs, promoting drug release, and improving brain selectivity.

Moreover, Liu et al. used an in vitro model to develop a new drug delivery system for LTG, a medication for epilepsy, using tryptophan-functionalized mixed micelles made of pluronic P123/F127 [109,110]. This delivery method was designed to overcome multidrug resistance by combining transporter-mediated endocytosis and pluronic block copolymer technology. The micelles effectively delivered LTG to the brain, especially the hippocampus, through tryptophan-mediated active targeting and modulation of P-gp (a protein involved in multidrug resistance) at the site of epilepsy.

Recently, Hou et al. proposed a dual-targeting nanocarrier system for delivering LTG to treat epilepsy. LTG had low solubility and a high risk of side effects due to its low bioavailability. The proposed delivery system consists of two components: D-form T7 peptide, which targets the blood-brain barrier, and Tet1 peptide, which targets neurons. The dual-targeting system was synthesized on a microfluidic chip using biodegradable poly(lactic-co-glycolic acid) to form a core and showed excellent therapeutic effects in both acute and chronic epilepsy models with high biosafety. This allowed LTG to pass across the BBB and then concentrate on the neuron. D-T7/Tet1-lipids@PL NPs exhibit good neuron targeting, anti-epileptic, and protective properties in both in vitro and in vivo tests [111]. This method offers a fresh method for enhancing the therapeutic potency of currently available anti-epileptic medications [112]. Other disorders of the central nervous system may also be treated with the help of this nanocarrier.

The use of new drug delivery systems has shown significant progress in the pharmacokinetic behavior and brain-targeting efficiency of LTG, becoming increasingly important in overcoming the limitations of traditional drug delivery methods, particularly in the context of multidrug resistance. Using a combination of active targeting and modulation of P-gp, these new systems can improve the efficacy and specificity of drug delivery to the target site, improve the efficacy of LTG in treating neurological disorders, reduce the risk of side effects, and improve patient outcomes.

### 2.2. Exploring the Relationship between LTG and Cancer as Potential Therapeutic Applications

Additionally, we checked the state of the art regarding LTG and its potential use in cancer research. LTG has been studied in preclinical models for the treatment of breast cancer and melanoma. LTG has shown promise as a potential treatment for breast cancer. In both in vitro and in vivo experiments, the drug was found to inhibit the growth of breast cancer cells, including hormone-resistant cell models, by regulating target genes of the FoxO3a transcription factor. LTG also increased the expression of PTEN and downregulated the PI3K/Akt signaling pathway, resulting in the activation of FoxO3a. The drug reduced tumor growth in vivo and increased FoxO3a expression, suggesting it could be a valuable option for targeted therapy in breast cancer patients [113]. In vitro and in vivo studies on tamoxifen-resistant breast cancer cells showed that the tamoxifen-resistant cells had low levels of FoxO3a, a protein that regulates cell growth and death, which was linked to their resistance to tamoxifen. However, re-expression of FoxO3a in these cells, either by induction with tetracycline or treatment with LTG, restored the sensitivity to tamoxifen and reduced the tumor mass in mice. Thus, LTG may be a promising combination therapy to prevent resistance to tamoxifen in patients [114].

Regarding skin cancer, a study conducted in Denmark found an association between the use of the anti-epileptic drugs carbamazepine and LTG and an increased risk of squamous cell carcinoma (SCC). The risk was enhanced by 59% in users of LTG compared to non-users, although with a less clear dose-response relationship [115]. The increased risk of skin cancer demonstrated in this study must be compared to the known advantages of anti-epileptic medication. It is important to stress that these findings must be replicated and characterized in different contexts.

A well-known fact is that both primary and metastatic brain tumors can cause seizures, although the frequency of seizures varies greatly depending on the kind of neoplasm. AEDs must be administered to patients with brain tumor-related seizures to avoid recurrence [116]. In 2016, a study aimed to assess the impact of anti-epileptic drugs on glioblastoma cell growth, including LTG. Results showed that LTG was not effective in inhibiting glioblastoma cell growth and had some growth-enhancing effects. LTG showed statistically significant growth inhibition in both cell lines; however, they did not reach IC50. The study concluded that LTG should be used as a treatment for epilepsy in glioblastoma patients with no risk of interfering with tumor development. Additionally, the study found that some anti-epileptic drugs, including valproic acid and oxcarbazepine, showed some efficacy in inhibiting glioblastoma cell growth; however, their use as monotherapy for glioblastoma treatment was ruled out due to high doses and low efficacy of the metabolites, respectively. The possibility of using these drugs as treatment is an area for future study [117]. In cancer patients, seizures are common and can be treated with AEDs. However, using AEDs together with chemotherapeutic drugs or tyrosine kinase inhibitors can result in drug interactions. Some newer AEDs, such as LTG, have minimal drug interactions, making them a good choice for treating seizures in cancer patients. LTG may have weak-inducing effects when combined with drugs metabolized by the same enzymes. Monitoring plasma drug levels is important to ensure proper treatment in patients receiving multiple drugs for cancer treatment [118].

LTG has effectively managed symptoms associated with cancer treatment and its related complications, such as epilepsy and seizures. However, limited research is available on the potential impact of LTG in treating cancer. It’s important to note that while these studies are promising, more research is needed to confirm the potential therapeutic use of LTG in cancer and other diseases [119,120]. Additionally, the safety and efficacy of LTG in these indications must be established through clinical trials. However, the results of these studies suggest that LTG may have a broader therapeutic potential than previously thought and may be a useful candidate for repurposing in the future. It is worth noting that LTG is currently not approved by regulatory agencies for cancer treatment. Further research is needed to determine the safety and effectiveness of LTG as a cancer treatment in humans.

## 3. Side Effects of Lamotrigine

Despite LTG’s widespread use, there are still significant limitations in our current understanding of its mechanism of action. One of the main limitations is the fact that LTG has multiple potential mechanisms of action in the brain. It still needs to be made clear which one or ones are primarily responsible for its therapeutic effects. Some studies suggest that LTG acts on neurotransmitter release, while others suggest that it modulates the activity of voltage-sensitive ion channels in the brain. Another limitation is that the specific neurobiological targets of LTG have yet to be fully characterized, making it difficult to understand exactly how the drug works at a molecular level. This lack of understanding also complicates the development of new and more effective treatments for neurological disorders.

This limited understanding also influences the confidence in dosing. There is still much to be learned about the optimal dosing of LTG for different patient populations and how its efficacy may be affected by the presence of comorbid conditions or the use of other medications [121,122]. Common CNS side effects of LTG can include drowsiness, headache, dizziness, and ataxia (uncoordinated movements). These side effects are usually mild and go away on their own, but in rare cases, LTG can cause serious CNS side effects such as seizures, confusion, and behavioral changes [123]. In some individuals, LTG can also cause a serious skin reaction known as Stevens-Johnson syndrome (SJS), which can be life-threatening. SJS can cause skin and mucosal tissue to peel off, and it can also cause severe eye damage [124]. SJS is believed to occur due to the accumulation of LTG in melanin-rich tissues. LTG and valproic acid interaction may cause SJS due to the inhibitory effect of valproic acid on the metabolism of LTG and the metabolism of valproic acid itself. SJS can be initiated by the formation of toxic metabolites via the minor metabolic pathway of LTG and the depletion of glutathione and L-carnitine [125]. Other factors contributing to the likelihood of SJS during therapy include genetic polymorphisms in the GSH and EPHX genes, food/drug allergies, recent viral illnesses, and adding antipsychotics to LTG treatment. To decrease the likelihood of SJS, it is recommended to supplement with GSH or L-carnitine, initiate LTG at a lower dose, and titrate more slowly, as well as conduct a search for genetic polymorphisms in relatives [126]. Understanding the inhibitory interaction between valproic acid and LTG can help practitioners stabilize LTG serum concentrations and adjust treatment regimens effectively [124]. It is well established that some genetic variables enhance the risk of SJS in response to LTG. Therefore, prior to commencing LTG, the Federal Food and Drug Administration advises testing HLA subtypes for those linked to SJS [124].

Investigating the mechanism of action by which LTG causes SJS could lead to a better understanding of the overall mechanism of action of the drug. A better understanding of the mechanism of action could inform the development of safer and more effective treatments for epilepsy and bipolar disorder, the conditions that LTG is used to treat. If the relationship between LTG accumulation in melanin-rich tissues and SJS is confirmed, it could lead to the development of better strategies to reduce the risk of SJS in individuals taking LTG. This could improve patient outcomes and increase the overall safety and efficacy of LTG as a treatment option [127].

Another potential side effect of lamotrigine that is not well understood is the risk of arrhythmia, which led the FDA to issue a warning in 2020 [128]. The exact mechanism by which lamotrigine may increase the risk of arrhythmia is not clear, but it may be related to the drug’s effects on ion channels and electrical signaling in the heart. LTG may affect the function of the HCN channels that are involved in regulating the heart rate. These effects on ion channels and HCN channels may contribute to changes in the electrical activity of the heart and increase the risk of arrhythmia [129,130]. It is important to note that the risk of arrhythmia with lamotrigine is still considered to be relatively low, and the drug is generally well tolerated by most patients. However, patients taking lamotrigine should be monitored closely for any signs of arrhythmia or other cardiovascular problems, especially if they have a pre-existing heart condition or are taking other medications that may interact with lamotrigine [131].

In summary, despite its widespread use and proven efficacy as a treatment for epilepsy and bipolar disorder, there are still significant limitations in our current understanding of the mechanism of action of LTG. Further research is needed to fully understand the drug’s mechanisms, as well as its interactions with other treatments and patient factors, to optimize its use for the treatment of neurological disorders.

## 4. Conclusions

LTG is a well-established anti-epileptic drug that has been shown to have potential as a repurposed treatment for other diseases of the CNS and cancer. Its ability to target voltage-gated sodium channels and modulate intracellular signaling pathways makes it a promising candidate for treating various diseases. Several preclinical studies have demonstrated its efficacy in both in vitro and in vivo models. Despite this promising research, clinical trials are still needed to confirm its safety and efficacy in the case of cancer patients.

However, the use of LTG as a treatment option for various neurological conditions, such as epilepsy and neuropathic pain, is limited by our current understanding of its mechanism of action. Despite its clinical efficacy, the exact mechanisms by which LTG confers therapeutic benefits remain elusive. This lack of understanding poses significant difficulties in optimizing its use in clinical settings and reducing the risk of adverse reactions. For example, the wide variability in the required dose of LTG for adequate pain relief highlights the difficulty in predicting the response to treatment and managing side effects. The onset and severity of common side effects such as sleepiness, dizziness, headache, vertigo, and ataxia can also vary greatly among patients, making it challenging to tailor their use to individual needs.

Furthermore, the potential for rare but serious adverse reactions such as Stevens-Johnson syndrome highlights the importance of further investigating the mechanisms underlying LTG’s therapeutic and adverse effects. A deeper understanding of the mechanisms of action of LTG would provide a more rational basis for its use, enabling more effective and safe treatment regimens to be developed. This will ultimately lead to more effective and safer treatments for patients suffering from neurological disorders and potentially for cancer treatment.

## Data Availability

Not applicable.
